# Using the Nonhuman Primate Model of HCMV to Guide Vaccine Development

**DOI:** 10.3390/v6041483

**Published:** 2014-03-27

**Authors:** Jesse D. Deere, Peter A. Barry

**Affiliations:** 1 Center for Comparative Medicine, University of California, Davis, Davis, CA 95616, USA; E-Mail: jddeere@ucdavis.edu; 2 Center for Comparative Medicine, Department of Pathology and Laboratory Medicine, California National Primate Research Center, University of California, Davis, Davis, CA 95616, USA

**Keywords:** cytomegalovirus, nonhuman primate, RhCMV, immune modulation, persistence, vaccine, reinfection

## Abstract

The natural history of human cytomegalovirus (HCMV) is inextricably associated with mucosal surfaces. The vast preponderance of primary infections occur following mucosal exposure to infectious virions, and the high seroprevalence of HCMV throughout the world is due to long-term excretion of HCMV in bodily fluids from multiple mucosal sites. Accumulating evidence presents a model where the earliest virus-host interactions following infection dictate the long-term pattern of infection, alter innate immune responses that skew adaptive responses to enable persistence within an immune host, and are essential for reinfection of a host with prior immunity. HCMV has evolved a complex repertoire of viral functions fine-tuned to manipulate the immune environment both locally at the sites of infection and systemically within an infected host. Collectively, viral immune modulation represents a significant impediment for an HCMV vaccine. As HCMV can disseminate beyond mucosal surfaces to reinfect immune hosts, it may not matter whether prior immunity results from prior infection or immunization. A better understanding of the earliest virus-hosts interactions at mucosal surfaces may identify elements of the viral proteome that are especially susceptible to vaccine-mediated disruption and prevent challenge virus from disseminating to distal sites, particularly the maternal-fetal interface.

## 1. Introduction

*Herpesviridae* are a family of ubiquitous pathogens with ancient origins, dating back 400 million years, and they have co-evolved with their radiating host species since the progenitor herpes virus species arose [1]. The extent of co-evolution is such that herpes simplex virus (HSV-1) sequences have been used to track human global migration out of Africa [2]. If evolutionary longevity and the percentage of infected hosts measure the success of a pathogen, this family is very successful. While the classification of *Herpesviridae* is based on virion structure, all herpes viruses share the phenotype of establishing a lifelong persistence within the infected and, usually, immune-competent host. Mechanisms of persistence remain an active area of research into herpes virus natural histories. While large gaps in our knowledge of persistence remain to be filled, it is becoming clear that there are common themes amongst all herpes viruses layered onto virus-specific functions that modulate host cell signaling, trafficking, activation, antigen presentation, and lifespan. Based on our current understanding, some viral immune modulation mechanisms can be sub-grouped by family (*alpha-*, *beta-*, or *gamma- herpesvirinae*) or can be species-specific. One unifying feature of all herpes viruses is the central role of epithelial cells in the infectious cycle in mediating the initial portal of entry during infection of an individual in addition to serving as the exit site for secretion into bodily fluid to maintain the virus within the larger population of susceptible hosts. In between these points of the HCMV infectious cycle, herpes viruses disseminate to family-specific cell types of persistence for the *alpha-, beta-, or gammaherpesvirinae*, including neurons, CD34^+^ and other cell types, and B cells, respectively. It is highly likely that the *raison d’être* for sites of persistence is to provide a privileged site for viral genomes, resistant to immune-mediated clearance, that can occasionally or subliminally reactivate to produce infectious virions that can traffic back to mucosal epithelial cells and be secreted in bodily fluids. Analysis of the central role of mucosal epithelial cells in the life cycle of human cytomegalovirus (HCMV) illustrates the complexities and the challenges confronting development of HCMV vaccines designed to prevent primary infection and, hopefully, reinfection of those with preexisting HCMV immunity.

## 2. HCMV Natural History

### 2.1. HCMV Shedding in Bodily Fluids

HCMV is the prototypical betaherpesvirus with a complex natural history, a large and incompletely defined genetic coding capacity, and a seemingly contradictory virus-host relationship. HCMV is ubiquitous throughout the world, and seroprevalence rates range from ~50% in the U.S. to close to 100% in some developing countries [3]. Primary HCMV infection in immune-competent individuals is generally associated with either transient and mild clinical outcomes or inapparent seroconversion. Based on the reported rate of congenital infection (~0.7%), primary infections result almost exclusively following mucosal exposure to horizontally transmitted virions in bodily fluids. Despite mucosal epithelia serving as the entry point for the preponderance of HCMV infections, there is virtually nothing known about the virological and immunological cascade of events within the mucosa and submucosa.

Like all herpes viruses, HCMV establishes a lifelong persistence characterized by the presence of cells harboring latent viral genomes that periodically reactivate to produce infectious virions. The frequency of shedding varies dependent in part on age, health, the particular bodily fluid, and physiologic status. In children under the age of five years infected congenitally or within the first years of birth, the frequency of HCMV in saliva and/or urine has been reported as high as 80% with rates declining as the children age (12%–23%) [4,5]. In healthy non-human immunodeficiency virus (HIV)-infected adults, cross-section surveys of HCMV excretion in cervical swabs, saliva, and/or urine indicate rates of detection between 7%–24%, with higher rates observed in the context of HIV coinfection (non-HAART treated) or sexually transmitted diseases [4,6,7,8,9]. Longitudinal studies of seroimmune healthy women demonstrate that 83% exhibited at least one positive urine sample with HCMV [8]. Rates of HCMV reactivation during pregnancy lead to the conclusion that HCMV is exquisitely suited for shedding in breast milk. Three studies have observed frequencies of HCMV detection in breast milk from 89%–100% [10,11,12]. While it is well established that HCMV can establish near complete latency in certain cell types (e.g., CD34^+^ myeloid progenitor cells), characterized by an extremely limited pattern of HCMV transcripts [13,14,15,16,17,18], long-term shedding in mucosal fluids is the norm during persistent infection. Even more pronounced than primary infection, persistent HCMV shedding is not associated with disease in those infected individuals with functional immune systems. As with the large gaps in our knowledge of initial virus-host interactions at mucosal surfaces during primary infection, the mechanistic basis for the frequency and magnitude of mucosal shedding are virtually unexplored for HCMV.

### 2.2. HCMV Immune Responses

The general absence of HCMV clinical sequelae associated with primary infection and the almost complete absence during persistent infection emphasize the protective nature of host antiviral immune responses. Primary HCMV infection stimulates robust immune responses, including broadly neutralizing antibodies to multiple envelope glycoproteins [19,20,21] and especially prominent antiviral T cell responses [22,23]. The protective efficacy of these immune responses is emphasized by the fact that declines in functional immunity, either by iatrogenic immunosuppression or immune deficiency, is associated with significantly increased risks of HCMV morbidity and mortality. Similarly, congenital HCMV infection is a leading infectious cause of hearing loss and developmental disorders in infants in the U.S. [21,24]. Primary HCMV infection in the mother puts the infant at greater risk of symptomatic disease [19]. Likewise, the immunological immaturity of fetuses portends increased risk of developmental deficits following congenital HCMV infection (reviewed in [25]). Due to the severe diseases that HCMV can cause in people with limited functional immunity, an HCMV vaccine has been a public health priority for the past several decades [24,26,27,28,29,30]. An overriding theme of HCMV vaccine development has been to recapitulate through vaccination the dominant immune responses generated during primary infection.

A potential limitation of this approach is that peripheral immune responses (e.g., neutralizing antibodies, antigen-specific T cells) constitute the vast majority of immune responses analyzed in infected individuals. While informative, limitation of immune responses to the periphery may result in failure to identify critical tissue-specific immune responses. In particular, mucosal immune responses *in situ*, including innate responses, to HCMV are notably understudied relative to peripheral B and T cell responses. A limited number of studies have detected very low but detectable binding and/or neutralizing antibody responses to HCMV in saliva following either natural infection and, importantly, immunization with live attenuated virus or recombinant gB subunit vaccination [31,32,33]. Saliva antibody responses, which are not detectable in all those with serum antibodies to HCMV, include IgG, IgA, and sIgA. Neutralizing antibodies have differentially been associated with the IgA and IgG fractions.

Three features of these studies that may be relevant to vaccine design include the following. (1) Vaccination can lead to detectable increases in saliva antibodies [33]; (2) Saliva IgG titers are a fraction of serum antibody IgG titers but generally correlate with peripheral IgG [31,33]; (3) There is no apparent association between cessation of HCMV shedding in saliva of young HCMV-infected children and development of detectable neutralizing antibodies in saliva [32]. As mucosal surfaces represent the first line of defense against HCMV infection, it stands to reason that a more thorough understanding of the earliest host-pathogen interactions at mucosal surfaces is critical for the future development of vaccine strategies designed to disrupt the natural pattern of infection, persistence, and shedding. Enhancement of innate immune responses at the site of infection might tip the balance in favor of the host and allow for viral clearance or reduced viral shedding from mucosal surfaces later in the replication cycle.

### 2.3. HCMV Persistence

The preceding discussion about the mostly protective immune responses resulting from primary infection presents something of a conundrum about the quality of the antiviral immune responses. Like all herpes viruses, HCMV persists for the lifetime of immune-competent hosts, and the longitudinal shedding of virus in bodily fluids demonstrates that viral gene expression occurs in the presence of the very same immune responses that protect against viral sequelae. Simply put, HCMV stimulates immune responses that effectively limit infection and mostly protect the host from disease, yet are insufficient to clear persistent reservoirs of infected cells. The chronicity of antiviral immunity following primary infection strongly implies chronic viral gene expression at a level sufficient to maintain antibody and T cell responses [23]. There is no solid evidence that lifelong HCMV infection and gene expression result in overt HCMV pathology, which leads to the conclusion that HCMV has evolved a lifecycle to maximize its potential to excrete infectious virions that can disseminate to susceptible hosts. Beyond the potential for spreading virus horizontally and vertically, there are numerous reports in the last several years suggesting that chronic HCMV infection may be associated with increased risk of some age-related diseases. It should be stressed, however, that the linkages are statistical associations without conclusive proof of HCMV causality [24,34]. It may turn out that, as human lifespan increases well beyond historic levels, chronic HCMV infection may have adverse effects in the context of an aging immune system.

### 2.4. HCMV Reinfection

Another aspect of the restricted immune protection conferred by primary infection is the failure of preexisting immunity to fully protect from reinfection with a different strain of HCMV [21,35,36]. Multiple studies have documented that HCMV can reinfect individuals with prior HCMV immunity [8,21,37,38,39,40,41,42,43]. Congenital infection rates of 1% have been reported in populations with 100% seroprevalence of HCMV. The demonstration that seropositive women who give birth to a congenitally infected infant acquired new antigenic reactivity to HCMV antigens between pregnancies is strong evidence that prior immunity is incompletely protective against reinfection with antigenic HCMV variants. This is particularly remarkable because, in healthy long-term HCMV carriers, ~10% of memory T cells are HCMV-specific, and neutralizing antibodies are generated against multiple viral glycoproteins including gB [23,44,45,46,47,48]. In another study, 30% of seropositive women that were longitudinally evaluated developed novel HCMV antibody specificities, equivalent to an annual rate of HCMV reinfection of 10% [49]. Studies of reinfection indicate a viral mechanism to surmount preexisting immune memory to initiate a new infection, leading to the clinical ramification that infection of an immune host is probably independent of whether prior immunity is from prior infection or prior vaccination. The reinfection studies also suggest that the true disease burden might be underestimated, particularly so since the reinfection studies to date are based on looking at an extremely limited number of epitope variants.

### 2.5. HCMV Immune Modulation

The ability of HCMV to persist in an immune-competent host and reinfect immune individuals is undoubtedly related to the extraordinary devotion of HCMV coding content to viral proteins that modulate host immune cell function. Based on the number of open reading frames that can be deleted without impairing replication in fibroblasts, it is likely that ~50% of viral ORF are involved in an extensive repertoire of immune modulating functions [50,51]. Given the protracted evolution of herpes viruses, in general, and HCMV, in particular, it is highly likely that evolution of viral functions that modulate innate and adaptive immunity define the mechanistic basis for the phenotypic features that define HCMV as the virus that it is. Consistent with this interpretation, it is, perhaps, noteworthy that there have been no descriptions of immune escape variants arising during subclinical persistent infections, or fulminant outcomes associated with immunosuppression or immunodeficiency. In contrast, drug-resistant variants readily arise after initiation of anti-HCMV chemotherapies [52], which, based on recent estimates of sequence variation occurring during HCMV infection, should come as no surprise [53]. One interpretation of these discordant responses to immune-mediated and antiviral-mediated selective pressures might be the result of the protracted evolution of herpes viruses in hosts possessing innate and adaptive immune systems. HCMV evolved the broad repertoire of immunomodulatory functions that presumably confers a selective advantage over genetic drift as a means to persist within a host and disseminate throughout the population. In contrast, anti-HCMV chemotherapies are very recent treatment modalities, and therefore, there are no apparent HCMV-encoded functions that can directly counteract the different anti-HCMV drugs in clinical use.

### 2.6. Future Directions for HCMV Vaccine Development

One of the earliest calls for development of an HCMV vaccine stemmed from the clinical recognition of the grave infectious threat that intrauterine HCMV represents to fetal growth and development [27]. Based on his 15-year retrospective analysis of congenital HCMV cases, Hanshaw concluded that, “*… any thoughtful program aimed at prevention or treatment deserves consideration*”. Multiple lines of reasoning, focusing on (1) lifelong viral gene expression and shedding; (2) the magnitude of host cell immune responses required to prevent sequelae; and (3) reinfection of immune hosts, lead to the argument that HCMV is unlike any other virus for which vaccines have been clinically approved. Accordingly, “any *thoughtful program aimed at prevention*” of HCMV infection is likely to require adjuncts to the current paradigm of preventing attachment of HCMV virions to susceptible cells by gB-mediated neutralization. There are numerous challenges confronting HCMV vaccine development including a complex natural history, incompletely defined correlates of immune protection, and financial and logistical challenges in sufficiently powered clinical trials. Recent studies using the rhesus macaque model of HCMV demonstrate that infection of rhesus macaques with rhesus CMV (RhCMV) offers novel insight into vaccine strategies that have clinical relevance for development of HCMV vaccines. The remainder of this review will focus on the mechanisms of RhCMV infection, transmission, persistence, and reinfection and how the results of these studies inform new HCMV vaccine modalities.

## 3. RhCMV Natural History

### 3.1. Endemic RhCMV Infectious Cycle in Rhesus Macaques (Macaca mulatta)

Development of the RhCMV model of HCMV persistence and pathogenesis has benefitted by characterization of RhCMV natural history in large breeding cohorts to establish normative parameters of endemic infections to guide experimental studies. One unifying element of population studies is the central role of mucosal surfaces in the infectious cycle of RhCMV. Studies have demonstrated that the kinetics of RhCMV dissemination throughout mixed cohorts of uninfected and infected animals is a function of the persistent shedding of virus in bodily fluids of infected animals and the repeated mucosal exposure of uninfected animals to virus. RhCMV is endemic in breeding cohorts of rhesus macaques, as well as in native populations of wild macaques, and almost 100% of naive animals seroconvert by one year of age, well before the onset of sexual maturity around four years of age [54,55,56,57]. There are no known clinical sequelae associated with primary RhCMV infection in immune competent animals, which stands in contrast to the severe pathogenic outcomes following SIV immunodeficiency [58,59,60] or iatrogenic immunosuppression [56]. Seroconversion of previously naïve animals is often, but not always, contemporaneous with the detection by TaqMan real-time PCR of low quantities of RhCMV genomes in plasma [61,62]. The quantities of viral DNA detected in plasma are lower than those detected in saliva later in infection, consistent with hematogenous spread of progeny virions from local portals of entry to distal sites throughout the body. Like HCMV, RhCMV can be detected in multiple cell types throughout the body [58,60], and the virus efficiently seeds those anatomic sites where virus can be shed.

Recapitulating the virus-host relationship for HCMV, RhCMV persists, and chronic RhCMV gene expression is the norm during the life of the infected animal, despite the presence of neutralizing antibody and T cell responses [63,64,65]. Key sites of persistence include the salivary glands and genitourinary tract. Infected animals can persistently and asymptomatically shed virus in bodily fluids (e.g., saliva and urine), often in high quantities, for years after primary infection [66,67,68,69]. Multiple genetic variants have been detected in fluids of naturally infected animals [69,70,71], although there are no reports of deep sequencing to quantify the extent of sequence diversity. Because of persistent shedding in bodily fluids and the social dynamics of large macaque cohorts, RhCMV rapidly spreads through the cohort. In fact, RhCMV has an exceedingly high force of infection in naïve hosts. Two studies of mixed cohorts of infected and uninfected macaques demonstrate that the doubling rate of seroconversion of uninfected animals is five to nine weeks [54,61].

There have been no reports to date of vertical transmission of RhCMV, possibly due to the universal seroprevalence of RhCMV in breeding age female macaques [56]. Consequently, the high seroprevalence of RhCMV in large cohort is the direct consequence of repeated mucosal exposure of uninfected animals to horizontally transmitted virions in bodily fluids of infected animals. As such, this aspect of RhCMV natural history reflects the challenges that confront HCMV vaccine trials, namely repeated close contact of vaccinated/uninfected women to those shedding potentially antigenically variant HCMV in bodily fluids. One implication from the studies of RhCMV in mixed populations is that there are at least two strategies to minimize the risk of primary infection: (1) vaccinate those at-risk for primary infection; and (2) vaccinate those who may become the source of horizontally transmitted virus to minimize the potential for shedding of virus in bodily fluids (such as young children). While detailed molecular and virological investigations of RhCMV infection following natural exposure have not been reported, experimental data regarding cellular tropism and the virally encoded genes that are essential in this process provide insights into parts of the viral proteome that may be especially susceptible to vaccine-mediated disruption.

### 3.2. Early Virus-Host Interactions during Experimental Infection

Early virus-host interactions within the mucosa following natural exposure to RhCMV have not yet been explored. However, several studies using experimental inoculation via subcutaneous (SC) delivery of virus have revealed a salient aspect about early virus-host interactions relevant to HCMV vaccine design. In particular, the acute virus-host interactions following SC inoculation determine the long-term patterns of RhCMV infection.

Several strains of RhCMV have been described in the literature, and strain-specific differences depend on the extent of fibroblast adaptation. RhCMV strain 68-1 is the prototypical strain that was isolated after co-culture of urine from a rhesus macaque on human fibroblasts [66,67]. Sequence analysis of the 68-1 genome after serial passage in human and/or rhesus fibroblasts revealed that the UL/b’ region of the genome had undergone rearrangements and loss of coding capacity, in particular RhUL128, RhUL130, and three CXC chemokine-like ORF [69,72]. Based on precedence with the HCMV gH-based pentamer [73,74], the loss of RhUL128 and RhUL130 results in severe attenuation of RhCMV tropism for epithelial and some endothelial cells in culture [75,76]. RhCMV strains UCD52 and UCD59 were also isolated initially on human and macaque fibroblasts, and subsequently passed exclusively on primary monkey kidney epithelial cells [61,77]. Sequence analyses of both UCD52 and UCD59 demonstrated that the UL/b’ regions of both strains retain the full coding capacity of wild-type RhCMV (*i.e.*, never passed in culture) [69], and both retain epithelial cell tropism *in vitro*. Of particular note for vaccine strategies, strain-specific differences in the UL/b’ coding content result in distinct phenotypes of acute infection *in vivo* following SC inoculation.

Skin biopsies of the SC inoculation site obtained seven days after infection with either UCD52 or UCD59 are noted for prominent neutrophilic infiltration with extensive infection of endothelial cells (CD31-positive), fibroblasts (vimentin-positive), and macrophages (CD68-positive) [60]. In contrast, biopsies from animals infected seven days prior with 68-1 were noted for a predominant mononuclear cell infiltrate, and infected cells were predominantly fibroblasts and a reduced infection frequency in macrophages was observed (compared to UCD52/UCD59). Notably, no endothelial cells infected with 68-1 were detected (infection of epithelial cells was not evaluated in this study). This study confirmed that the expanded tropism conferred by the gH-anchored pentamer (in UCD52 and UCD59) observed *in vitro* is also observed *in vivo*. In addition, investigation of the earliest virus-hosts interactions highlights how RhCMV acutely alters local innate host responses, presumably due to the presence (UCD52/UCD59) or absence (68-1) of viral CXC chemokine-like ORF. As will be discussed below (Section 3.3 and Section 3.4), differences in UL/b’ coding capacity also result in distinct long-term parameters of RhCMV infection.

### 3.3. Rapid Emergence of RhCMV Variants with Complete UL/b’ Coding Capacity during Experimental Infection

The strong positive selective pressures conferred by a full complement of UL/b’ coding capacity was recently described in a serendipitous finding with another annotated strain of RhCMV [78]. RhCMV strain 180.92 was isolated from a simian immunodeficiency virus (SIV)-infected monkey and serially passed on human and monkey fibroblasts without plaque purification [79]. Sequence analysis of overlapping cosmid clones generated from cells infected with the 180.92 stock revealed a large rearrangement in UL/b’ that resulted in the deletion of most of UL/b’-encoded ORF, compared to 68-1 and wild-type RhCMV, but left intact the RhUL128-131 coding regions. RhCMV 180.92 exhibits greater epithelial/endothelial cell tropism than 68-1, consistent with retention and expression of the complete gH-anchored pentamer in 180.92, although the ability to infect epithelial/endothelial cells is reduced compared to a repaired version of 68-1 (BRh68-1.2) in which the RhUL128 and RhUL130 genes were engineered back into 68-1 [75]. Reduced growth of 180.92 in epithelial/endothelial cells compared to BRh68-1.2, is likely due to other UL/b’ ORF distinct from RhUL128-131. Stocks of RhCMV 180.92 were prepared from bulk infected cell cultures, and plaque purification was never done during serial passage. PCR analysis of the 180.92 stock subsequently revealed the presence of a minor sequence variant comprising <15% of the total RhCMV genomes within the 180.92 stock. PCR amplification and sequencing of molecular clones determined that the minor sequence variant was, in fact, a full-length RhCMV genome, presumably representing the remnant of the original unrearranged genome prior to fibroblast adaptation. Reexamination of tissues from these animals and other animals unexpectedly revealed that there was a rapid emergence of the full-length variant despite it being a minor constituent in the virus stock [78].

Six RhCMV uninfected macaques, including one that was SIV-infected, were inoculated intravenously with the RhCMV 180.92 stock, and longitudinal plasma, saliva, and urine samples were collected and analyzed for RhCMV. Using primer sets that distinguished the truncated and full-length genomic variants, it became apparent that the full-length variant rapidly became the dominant genomic form (>95%) in blood by three weeks post inoculation. Analysis of RhCMV in the tissues from the SIV/RhCMV coinfected animal similarly demonstrated high-level genome copy numbers of the full-length in tissues. In contrast, the truncated genomic variant exhibited significantly lower genome copy numbers in plasma, tissues, urine, and saliva. These data demonstrate that the truncated 180.92 variant is severely attenuated *in vivo*, despite the presence of the RhUL128-131 ORF. The results emphasize that additional UL/b’ ORF are essential for optimal replication and dissemination of RhCMV *in vivo*, and accordingly, these particular viral proteins represent potential vaccine targets to minimize hematogenous spread to tissues throughout the body.

### 3.4. Long-term Parameters of RhCMV Infection during Experimental Infection

The preceding rationale about vaccine-mediated targeting those UL/b’ ORF outside of RhUL128-131 can also be applied to RhUL128-131, based on differences in long-term infections of variants containing or lacking RhUL128-131. In addition to an apparent defect in cell tropism during acute infection (described above), 68-1 is significantly attenuated for shedding in bodily fluids [60,61]. Uninfected animals inoculated SC with UCD52 and UCD59 are noted for persistent detection of RhCMV in the saliva and urine of most animals (~75%) beginning six to eight weeks after inoculation [61]. The prolonged detection of RhCMV following experimental inoculation reflects the pattern of RhCMV shedding in animals naturally exposed to RhCMV [66,67,68]. In marked contrast, 68-1 DNA is either below the limit of PCR detection, or infrequently detected at low copy numbers in either bodily fluid [80,81,82]. All of these studies used PCR detection of RhCMV DNA as a surrogate for virus isolation to assess virus shedding. As a measure of the biological relevance of PCR detection, animals inoculated with UCD52 or UCD59 can transmit virus to uninfected cage-mates, whereas horizontal transmission of 68-1 has not been observed [61]. This finding is particularly relevant for vaccine development strategies aimed at reducing viral shedding as a means to control horizontal infection. Together, these observations suggest impaired dissemination and shedding of 68-1 *in vivo* and demonstrate the essential nature of mucosal tissues in CMV infection and the requirement for broad cellular tropism. It should be noted that RhCMV 68-1 is not replication impaired *in vivo* due to the absence of a functional gH-anchored pentamer and the CXC-chemokine-like ORF. The pattern of acute infection with 68-1 (described in Section 3.2) [60] demonstrates that 68-1 can utilize, presumably, gB-mediated entry into permissive cells, and 68-1 can disseminate throughout the body [83]. Moreover, studies using RhCMV 68-1 as a vaccine vector to express SIV antigens have observed some excretion of the engineered 68-1 vectors in urine of inoculated animals [22,84].

Although the mechanisms involved in mucosal transmission are poorly understood, disruption of the natural pattern of mucosal shedding through vaccination, by either reducing the incidence of shedding in infected people or enhancing mucosal immune responses to prevent infection or reinfection, is a novel strategy that is supported by recent observations in macaques. A better understanding of the early interactions between RhCMV and its host, and the mucosal immune responses involved in RhCMV infection, are essential for this vaccine development strategy.

### 3.5. RhCMV Immune Modulation

It is an accepted precept of vaccinology that the quality and quantity of adaptive immune responses are dependent on the quality and quantity of innate responses [85]. Accordingly, there is increasing effort in designing adjuvants that direct adaptive immunity in a direction that increase protective efficacy. Acute differences in innate immune responses at sites of inoculation following SC exposure to either UCD52/UCD59 or 68-1 (described above) [60] imply that RhCMV (and by extension HCMV) similarly exerts an adjuvant-like effect on the local innate effector cells to direct adaptive immune responses towards a situation that favors viral persistence at the expense of immune-mediated clearance of RhCMV-infected cells. The RhCMV functions that presumably mediated the relative differences in the nature of the inflammatory response (polymorphonuclear cells for UCD52/UCD59 and mononuclear cells for 68-1) is due to the presence of the CXC-like ORF in UCD52/UCD59, although no functional activity for these ORF has been reported [69]. The concept that a critical step in early RhCMV-host interactions involves rapid manipulation of innate responses is bolstered by studies investigating the role of the virally encoded interleukin-10 like protein of RhCMV.

Primate CMVs, with the exception of chimpanzee CMV, encode a viral IL-10 gene that is an evolutionary remnant of a transduced cellular IL-10 (cIL-10) gene by a progenitor primate CMV at some point after the split of rodents and primates [56,86,87]. Despite extensive genetic drift of the HCMV and RhCMV viral IL-10 proteins (cmvIL-10 and rhcmvIL-10, respectively) from the cIL-10 proteins of their respective hosts, these viral orthologs retain high affinity for the cognate cIL-10 receptor [50] and nearly identical immunosuppressive properties *in vitro* to those exhibited by cIL-10 [56,87]. The conservation of functionality of cmvIL-10 and rhcmvIL-10 to that of cIL-10 leads to the proposition that expression of an IL-10-like protein in the context of viral infection suppresses acute immune responses to viral infection.

The role of rhcmvIL-10 in primary infection was analyzed in macaques following SC inoculation with 68-1 or a 68-1 rhcmvIL-10 knockout virus (68-1ΔIL-10) [81]. Tissue biopsies taken from animals seven days after inoculation with 68-1 had markedly reduced cellularity at the inoculation site, but greater numbers of tissue macrophages, than 68-1ΔIL-10 inoculated animals [81]. In addition, there were fewer myeloid dendritic cells and decreased priming of naïve CD4^+^ T cells in the draining lymph nodes of 68-1 infected animals *versus* 68-1ΔIL-10 infected animals. Animals inoculated with 68-1 also showed impaired RhCMV-specific IgG responses and lower IgG avidity, compared with 68-1ΔIL-10 infected animals. In sum, the functional absence of rhcmvIL-10 in 68-1ΔIL-10 resulted in increased innate immune recognition of viral antigens and increased adaptive immune responses. These results lead to a model whereby a critical step in RhCMV infection is the immediate manipulation of the local immune environment mediated, in part, by rhcmvIL-10. Based on this premise, secreted proteins, such as rhcmvIL-10, should represent reasonable vaccine targets to induce, particularly, antibodies that neutralize their function.

A recent survey of RhCMV-infected macaques revealed that one consequence of the extensive genetic drift of rhcmvIL-10 from the cIL-10 protein of its host is that what was once a self-protein soon after transduction of the cIL-10 gene has become highly immunogenic in the context of viral infection [88]. All RhCMV-infected animals develop high avidity binding antibodies to rhcmvIL-10, and almost 100% of the animals also exhibit some level of antibodies that neutralize rhcmvIL-10 function in bioassays. Importantly, the rhcmvIL-10-specific neutralizing antibody responses generated in these macaques did not cross-react with cIL-10. The cmvIL-10 protein is also immunogenic in HCMV-infected humans [89]. Together, these data provide proof-of-concept for targeting viral IL-10, an immune modulator, as a vaccine strategy to interrupt viral persistence.

To accomplish antigenic stimulation without triggering the cIL-10 receptor, a non-functional rhcmvIL-10 has been designed, cloned into an expression cassette, expressed, and purified [90]. Immunization of naïve macaques with a DNA prime/protein boost immunization strategy stimulated antibody responses that neutralized wild-type rhcmvIL-10 function without cross-neutralization of rhesus cIL-10 [90]. SC challenge of vaccinated animals with UCD59 resulted in significantly lower frequencies and loads of RhCMV DNA in plasma, saliva, and urine, compared to mock-vaccinated control animals [91]. These data are unprecedented for CMV vaccine strategies in that targeting a single viral immune modulator (*i.e.*, rhcmvIL-10) elicited immune-mediated protection against viral challenge without inducing antibodies that neutralized RhCMV infection of cells. Given the large devotion of the HCMV coding capacity to immune modulating proteins (discussed in Section 2.5), the results targeting rhcmvIL-10 offer a compelling rationale to explore additional viral antigens involved in immune modulation.

### 3.6. RhCMV Reinfection

A considerable challenge to reducing congenital infection is the ability of HCMV to reinfect women with pre-conceptional immunity and subsequently cross the maternal-fetal interface (discussed in Section 2.4). The challenge arises because the mechanism for reinfection is unknown and, consequently, there are no prevention strategies short of absolutely minimizing exposure to virus shedding contacts. Fortunately, a study of RhCMV sheds light on viral mechanisms to overcome prior immunity.

Evidence for RhCMV infection of immune competent hosts is suggestive from natural history studies but incontrovertible from experimental studies. As described above (Section 3.1), multiple genetic variants of RhCMV have been amplified by PCR from RhCMV-immune animals. While these reports are consistent with reinfection, given the repeated exposure to infectious virus in bodily fluids of infected cohorts, it cannot be ruled out that the animal, from which saliva samples were analyzed, was not infected with more than one variant during primary exposure to RhCMV. In contrast, experimental inoculation of immune animals with genetically tagged variants of RhCMV is exceedingly efficient. Engineered variants of RhCMV 68-1 that express the SIV proteins (gag, retanef) have been used to reinfect RhCMV immune animals [84]. Animals inoculated with the RhCMV vector expressing the SIV protein uniformly develop SIV protein-specific T cells responses, and the RhCMV/SIV vector can be recovered in the urine of reinfected animals. Reinfection can be observed following inoculation with as little as 100 PFU, highlighting the ability of RhCMV to overcome existing antiviral immune responses [22].

Based on this, Hansen *et al.* reasoned that an essential step in reinfection is overcoming “*an initial immunological checkpoint*”, and they addressed whether the RHCMV orthologs of the HCMV proteins disrupting MHC-I antigen presentation mediate RhCMV reinfection (US2, 3, 6, and 11) [22]. Engineered deletion of the RhUS2-11 genes did not impair the ability of the deletant variant to replicate in cultured fibroblasts, compared to the unmodified parental vector. RhCMVΔUS2-11 expressing SIV gag was also able to establish infection in RhCMV-uninfected macaques, as demonstrated by recovery of RhCMVΔUS2-11 in the urine of infected animals and development of RhCMV-specific and gag-specific T cell responses. Notably, RhCMVΔUS2-11 was incapable of reinfecting RhCMV-immune animals following SC inoculation with 10^7^ PFU. These results demonstrate that manipulation of the host immunity is essential for reinfection, and, together with studies of acute RhCMV infection, illustrate the role RhCMV immune modulating proteins play in the infectious cycle of RhCMV *in vivo*.

## 4. Conclusions

The results of the Phase II gB vaccine trial [29] represents an important step towards meeting the clarion call of Hanshaw to develop a vaccine that protects against the devastating consequences of intrauterine HCMV infection [27]. This clinical trial now represents the “gold standard” against which subsequent HCMV vaccine trials will be compared, and yet, it is not certain at this time whether the level of observed protection compels a Phase III trial. Key issues about how best to move forward include (1) optimization of gB immunization to induce greater protective neutralizing responses; (2) inclusion of additional envelope glycoproteins to induce broader neutralizing responses against other virologically relevant cell types (*i.e.*, gH pentamer-mediated infection of epithelial and endothelial cells); and/or (3) targeting other parts of the HCMV proteome not involved in attachment/entry of susceptible cells. The vaccine potential for broadening neutralizing antibody responses beyond gB has been demonstrated recently by restoring the gH pentameric complex in the AD169 vaccine strain of HCMV [92,93]. While still in an early developmental stage, this strategy may help to address the difficult problem of the broad cellular tropism of HCMV. HCMV persistence in immune competent hosts and, in particular, the ability of HCMV to reinfect immune individuals leads to the proposition that HCMV is unlike most every other virus for which protective vaccines have been developed. Varicella zoster virus (VZV), an alphaherpesvirus and the only herpes virus for which there is a commercially available vaccine, persists in the infected host in a truly latent state, and recurrent disease is the result of reactivation of this latent virus after host immune responses have waned or become impaired. The attenuated VZV vaccine strain, Oka, was generated via serial passage in cell culture of a clinical VZV isolate. Similar strategies for HCMV have failed to generate a successful vaccine candidate (e.g., Towne) [94,95]. In addition, prior immunity to HCMV does not protect from reinfection despite robust adaptive immune responses. The ability to overcome extant neutralizing antibody responses and extraordinarily large HCMV-specific T cell responses emphasize the unprecedented role of HCMV-encoded immune modulating functions in HCMV natural history. Given the expense involved in conducting sufficiently powered clinical Phase II and Phase III trials, there is an essential need for rigorous pre-clinical studies that can inform which vaccine modalities should advance to human testing.

Recent studies of RhCMV point to multiple ORF that can be targeted to change the course of acute infection, dissemination via the blood to tissues throughout the body, and the ability to seed distal sites where progeny virions are shed into bodily fluids. These antigens include: gB, pp65, the gH-pentamer complex, CXC chemokines, rhcmvIL-10, and other ORF in UL/b’ besides RhUL128-131. However, multiple RhCMV vaccine studies using a variety of antigen delivery modalities have demonstrated partial protection, at best, and the results are consistent with the complexities of HCMV vaccine design. It should be stressed that all RhCMV vaccine studies, and to our knowledge all animal vaccine models of HCMV vaccines, use direct parenteral introduction of challenge virus. This bypasses the potential for mucosal immunity in the vaccinee to control primary viral challenge, and subsequent studies in animal models should work to recapitulate the repeated mucosal exposures to HCMV that vaccinees will encounter.
